# A novel aspirin prodrug inhibits NFκB activity and breast cancer stem cell properties

**DOI:** 10.1186/s12885-015-1868-7

**Published:** 2015-11-04

**Authors:** Irida Kastrati, Vladislav A. Litosh, Shuangping Zhao, Manuel Alvarez, Gregory R. J. Thatcher, Jonna Frasor

**Affiliations:** 1 Department of Physiology and Biophysics, University of Illinois at Chicago, 835 S. Wolcott, E202 MSB, MC901, Chicago, IL 60612 USA; 2 Department of Medicinal Chemistry and Pharmacognosy, University of Illinois at Chicago, Chicago, IL 60612 USA

**Keywords:** Breast cancer, Aspirin, Cancer stem cells, Fumarate, NFκB

## Abstract

**Introduction:**

Activation of cyclooxygenase (COX)/prostaglandin and nuclear factor κB (NFκB) pathways can promote breast tumor initiation, growth, and progression to drug resistance and metastasis. Thus, anti-inflammatory drugs have been widely explored as chemopreventive and antineoplastic agents. Aspirin (ASA), in particular, is associated with reduced breast cancer incidence but gastrointestinal toxicity has limited its usefulness. To improve potency and minimize toxicity, ASA ester prodrugs have been developed, in which the carboxylic acid of ASA is masked and ancillary pharmacophores can be incorporated. To date, the effects of ASA and ASA prodrugs have been largely attributed to COX inhibition and reduced prostaglandin production. However, ASA has also been reported to inhibit the NFκB pathway at very high doses. Whether ASA prodrugs can inhibit NFκB signaling remains relatively unexplored.

**Methods:**

A library of ASA prodrugs was synthesized and screened for inhibition of NFκB activity and cancer stem-like cell (CSC) properties, an important PGE2-and NFκB-dependent phenotype of aggressive breast cancers. Inhibition of NFκB activity was determined by dual luciferase assay, RT-QPCR, p65 DNA binding activity and Western blots. Inhibition of CSC properties was determined by mammosphere growth, CD44^+^CD24^−^immunophenotype and tumorigenicity at limiting dilution.

**Results:**

While we identified multiple ASA prodrugs that are capable of inhibiting the NFκB pathway, several were associated with cytotoxicity. Of particular interest was GTCpFE, an ASA prodrug with fumarate as the ancillary pharmacophore. This prodrug potently inhibits NFκB activity without innate cytotoxicity. In addition, GTCpFE exhibited selective anti-CSC activity by reducing mammosphere growth and the CD44^+^CD24^−^immunophenotype. Moreover, GTCpFE pre-treated cells were less tumorigenic and, when tumors did form, latency was increased and growth rate was reduced. Structure-activity relationships for GTCpFE indicate that fumarate, within the context of an ASA prodrug, is essential for anti-NFκB activity, whereas both the ASA and fumarate moieties contributed to attenuated mammosphere growth.

**Conclusions:**

These results establish GTCpFE as a prototype for novel ASA-and fumarate-based anti-inflammatory drugs that: (i) are capable of targeting CSCs, and (ii) may be developed as chemopreventive or therapeutic agents in breast cancer.

**Electronic supplementary material:**

The online version of this article (doi:10.1186/s12885-015-1868-7) contains supplementary material, which is available to authorized users.

## Background

Inflammation is a well-established cancer risk factor that affects incidence, promotion, and progression and is widely associated with an overall poor patient outcome [1, 2]. In breast cancer, epidemiological studies report an inverse association between the use of non-steroidal anti-inflammatory drugs (NSAIDs) and breast cancer risk [3–5]. In particular, regular use of the classical NSAID, aspirin (acetylsalicylic acid, ASA), leads to a reduction in breast cancer incidence [6–9]. Although there is a general consensus on the benefits of aspirin use, a limited number of studies, such as the study by Cook et al. [10], reported no such benefits. These inconsistencies may be reconciled if aspirin dose, duration, and study design are taken into consideration. The anti-cancer effects of ASA are primarily attributed to its ability to inhibit cyclooxygenase 2 (COX2) activity, which is often up-regulated in breast cancer [11, 12], and reduce production of prostaglandin E2 (PGE2), the predominant secreted prostaglandin in breast tumors [13]. A number of studies suggest that ASA may also act, at least in part, by suppressing aberrant nuclear factor κB (NFκB) signaling [14–18]. This activity would be desirable in breast cancer since NFκB can promote tumor cell survival, proliferation, migration, invasion, angiogenesis, and resistance to therapy [19–21].

More recently, both the COX2/PGE2 axis and the NFκB signaling pathway have been implicated in the survival and propagation of breast cancer stem cells (CSCs) [22–28]. According to the CSC hypothesis, breast CSCs are a subset of cells within the tumor that can self-renew, differentiate, and evade anoikis [29–31]. CSCs are also highly tumorigenic, therapy resistant, and involved in metastasis and tumor recurrence [32–37]. Therefore, it is thought that successfully targeting breast CSCs may sensitize resistant tumors to therapy and prevent future recurrence and metastasis. Moreover, it is plausible that anti-inflammatory drugs that simultaneously target both the COX2/PGE2 and NFκB pathways, such as ASA, can be exploited to eradicate CSCs.

Unfortunately, the use of ASA to achieve COX2 and NFκB inhibition is associated with gastrointestinal (GI) toxicity [38]. Even at the lowest dose of daily use (81 mg/baby aspirin), ulcers and stomach bleeding occur and exemplify the limitations of extended ASA use [39]. For NFκB inhibition, the problem is compounded further by the low potency of ASA on this pathway. For example, the lowest reported IC_50_ for inhibition of IKKβ, a key kinase in the NFκB pathway, by ASA is 80 μM on purified protein *in vitro* [15]. In cells or animal models, the dose of ASA required to inhibit NFκB is a thousand-fold higher [14–18]. To overcome GI toxicity, ASA prodrugs have been developed, validated in animal models, and advanced to clinical trials [40–44]. The prodrug strategy consists of converting ASA into an ester prodrug, thereby introducing lipophilicity into the molecule and masking the carboxylate’s hydrogen bonding groups. In turn, this enhances cellular uptake and permeability of ASA prodrugs. The resulting enhanced potency allows for reduced doses, which then minimizes GI toxicity. To further enhance potency and/or add functionality, design of prodrugs may also incorporate other structural elements or ancillary pharmacophores.

**Fig. 1:**
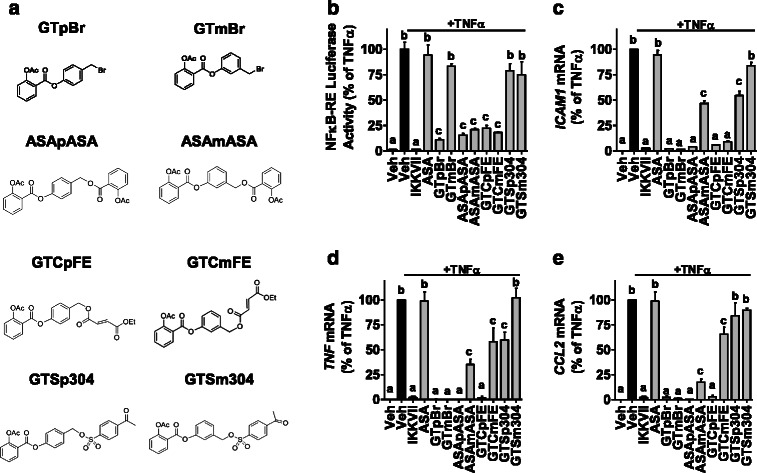
ASA prodrugs inhibit NFκB activity in breast cancer cells. **a** Chemical structures of ASA prodrugs are indicated. Four ancillary pharmacophores, bromide (Br), acetylsalicylate (ASA), fumarate (FE), or sulfonate (S), were incorporated in either *para* (p) or *meta* (m) position. **b**-**e** MCF-7 cells were pretreated for 2 hours with 1 μM of IKKVII, 50 μM of ASA, 50 μM of ASA prodrugs, or vehicle (Veh) followed by treatment with TNFα (10 ng/ml) for 2 hours. **b** NFκB-RE activity was measured by dual luciferase reporter assay after TNFα (10 ng/ml) for 4 hours. (**c**-**e**) Expression of NFκB target genes, ICAM1, TNF and CCL2 was measured by RT-QPCR. Drug inhibitory activity is plotted as % of TNFα alone. Data points with different letters (a, b, c) are significantly different from one another, *P* < 0.05

While ASA prodrugs have been studied as COX inhibitors, their specific activity on the NFκB pathway in breast cancer remains relatively unexplored [45, 46]. To address this, we synthesized a series of ASA ester prodrug pairs that incorporate ancillary pharmacophores, some with proven anti-inflammatory activity, in either *para* (p) or *meta* (m) position (Fig. 1a) [47–53]. The objective was to identify prodrugs with enhanced potency for NFκB inhibition, reduced cytotoxicity, and selective targeting of breast CSCs, which together would indicate a favorable therapeutic index. While several of these ASA prodrugs are potent NFκB inhibitors, they are also cytotoxic. In contrast, GTCpFE, a fumarate-based ASA prodrug, is an effective NFκB inhibitor without any concomitant cytotoxicity. Moreover, GTCpFE can effectively target breast CSCs by simultaneously inhibiting both COX and NFκB pathways. As a consequence, this prodrug strategy lays the groundwork for future anti-inflammatory and anti-CSC drug development.

## Methods

### Reagents

TNFα and IL-1β were purchased from R&D Systems. ASA was purchased from Sigma. IKKVII was purchased from EMD Millipore. Antibodies for p-IKKα/β (#2697), IKKα (#2682), IKKβ (#2370), p-IκBα (#2859), IκBα (#4814) and p-p65 (S536, #3033) were purchased from Cell Signaling. The antibody for p65 (sc-372) was purchased from Santa Cruz and β-actin (A5441) from Sigma.

### ASA prodrugs synthesis

GTpBr, GTmBr, and ASApASA were synthesized as previously reported [42, 47]. ASAmASA, GTCpFE, GTCmFE, GTSp304, GTSm304, BzFE, and GTCpSE were synthesized, purified, and fully characterized as described in the Additional file 1: Supplementary Methods and Additional file 2: Figure S1.

### Cell lines and culture conditions

Well characterized human cell lines that are genetically and phenotypically different but represent major breast cancer subtypes were used for these studies. For the luminal estrogen receptor (ER) + subtype, we utilized MCF-7 and T47D cells, which express high levels of ER and proliferate in response to estrogen treatment. For the HER2 subtype, we utilized BT474 cells, which overexpress the oncogene epidermal growth factor receptor 2 (Her2). For the triple negative subtype, we utilized MDA-MB-231 cells, which are basal/mesenchymal cell types, and lack expression of ER, PR and Her2. MCF-7, T47D, and BT474 cells were obtained from Dr. Debra Tonetti (University of Illinois at Chicago). These cells were routinely maintained in RPMI 1640 media (Invitrogen Life Technologies) with phenol red supplemented with 10 % FBS, 1 % non-essential amino acids, 2 mmol/L L-glutamine, 1 % antibiotics penicillin-streptomycin, and 6 ng/mL insulin. MDA-MB-231 cells were obtained from Dr. Clodia Osipo (Loyola University Chicago) and routinely maintained in IMEM media (Corning) supplemented with 5 % FBS, 1 % non-essential amino acids, 2 mM L-glutamine, and 1 % antibiotics penicillin-streptomycin.

### Luciferase reporter assay

MCF-7 cells were transiently co-transfected with an NFκB-RE luciferase construct (Clontech) along with the renilla luciferase construct, pGL4.70 (Promega), and dual luciferase assays were carried out as previously described [54].

### RT-quantitative PCR (QPCR)

Total RNA was isolated using the Trizol method, then reverse transcribed (RT), and analyzed by QPCR performed as previously described [55]. Fold change was calculated using the ΔΔ*C*t method with 36B4 serving as the internal control. QPCR primer sequences are available upon request.

### p65 DNA binding assay

Nuclear extracts were isolated and p65 DNA binding activity was measured via an ELISA (Active Motif) according to manufacturer’s guidelines.

### Western blot

Whole cell extracts were prepared using the M-PER reagent (Thermo Scientific). Proteins were separated by SDS-PAGE (Bio-Rad Laboratories), transferred to nitrocellulose membranes (Thermo Scientific), blocked for 1 hour in buffer containing 5 % nonfat dry milk (Lab Scientific) or 5 % bovine serum albumin, and incubated with the appropriate primary antibody overnight. The next day, secondary antibody was applied and the signal was visualized on a Molecular Imager ChemidocXRS (Bio-Rad Laboratories) using the Pierce Supersignal West Pico chemiluminescent substrate (Thermo Scientific). Images were obtained using Quantity One software (Bio-Rad Laboratories).

### MTS viability assay

Cell viability upon drug treatment was measured via the CellTiter96® AQueous One Solution assay (Promega).

### Mammosphere (MS) assay

Breast cancer cells were seeded at single cell density on low attachment plates in media described by Dontu et al., supplemented with 1 % methyl cellulose to prevent cellular aggregation [29]. After 7 days, the diameter of MS was measured and MS ≥75 μm in diameter were counted. For MS formation studies, inhibitors were added the day after seeding. For RNA, p65 DNA binding activity, and protein studies, MS were grown for 7 days and inhibitors were added for the last 3–6 hours.

### PGE2 assay

For measuring secreted PGE2, conditioned media was collected after 24 hours of treatment and a PGE2 ELISA (R&D Systems) was run according to the manufacturer’s specifications.

### CSC immunophenotype

Antibodies for CD44 and CD24 were purchased from Pharmingen. Cell labeling and flow cytometry was done according to Liu et al. [56].

### Tumorigenicity in athymic mice

All mouse experiments were carried out at the University of Illinois at Chicago animal facility. All mouse experiments were conducted in accordance with institutional procedures and guidelines, and prior approval from the Institutional Animal Care and Use Committee. Female athymic nude mice (nu/nu), aged 4–5 week-old, were purchased from Harlan. Following 72 hour pretreatment with DMSO vehicle (Veh) or GTCpFE, one million MDA-MB-231 cells were injected orthotopically into the thoracic mammary glands (N = 8 injections per group). Tumor formation was monitored by palpitation and day 1 was considered the first day a tumor was observed. Tumor size was then measured 3 times per week with an electronic caliper.

### Statistical analysis

Data are presented as mean ± SEM from at least three independent determinations. Statistical analysis consisted of 1- or 2-way ANOVA followed by Tukey posttest, or *t* test, as appropriate.

## Results

### Anti-NFκB activity of aspirin prodrugs in breast cancer cells

**Fig. 2:**
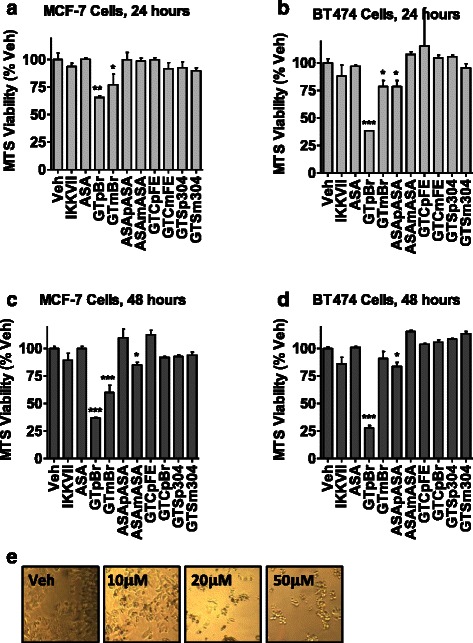
Effect of ASA prodrugs on cell viability. **a**-**d** MCF-7 (A, C) or BT474 (B, D) cell viability using the MTS assay was measured after 24 or 48 hours of treatment with 1 μM of IKKVII, 10 μM of ASA or 10 μM of ASA prodrugs. Drug activity is plotted as % of DMSO vehicle control. Stars above indicate significance compared to control, * *P* < 0.05, ** *P* < 0.01, *** *P* < 0.001. **e** Representative pictures of MCF-7 cells after 24 hours treated with increasing concentrations of ASApASA indicate an abnormal cell phenotype

To determine whether the ASA prodrugs we synthesized (Fig. 1a and Additional file 2: Figure S1) inhibit the NFκB pathway, their activity was screened in MCF-7 breast cancer cells at one dose (50 μM) on NFκB-RE and NFκB target gene endpoints (Fig. 1b-e). The pro-inflammatory cytokine, TNFα, was used to activate the NFκB pathway and IKKVII, a known IKKα/β inhibitor, was used as a positive control. We find that the ASA prodrugs incorporating bromide, acetyl salicylate, and fumarate but not sulfonate or ASA itself, significantly inhibit both NFκB-RE activity and NFκB target genes, including ICAM1, CCL2, and TNF. To determine the therapeutic potential of these ASA prodrugs, we next examined whether they were cytotoxic. Both the bromide and acetylsalicylate analogs significantly reduce cell viability of MCF-7 cells and a second breast cancer cell line, BT474 (Fig. 2). However, the known NFκB inhibitor, IKKVII, or ASA itself do not show the same effect on cell viability. This suggests that the bromide and acetyl salicylate prodrugs, besides NFκB inhibition, display additional off-target activity. Therefore, the bromide and acetyl salicylate prodrugs were withdrawn from further consideration. This, in combination with sulfonates’ poor NFκB pathway inhibition, led to the fumarate pair emerging as the best candidates, and GTCpFE was selected as a prototype for further detailed study.

**Fig. 3:**
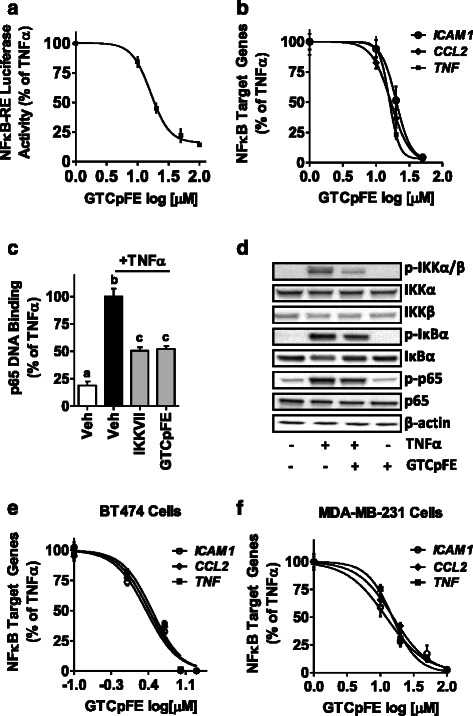
GTCpFE inhibits TNFα-induced NFκB signaling. **a** TNFα-induced NFκB-RE activity and **b** NFκB target gene expression was measured in MCF-7 cells pretreated with increasing concentrations of GTCpFE. IC_50_s were calculated using GraphPad software. **c** p65 DNA binding activity was measured in MCF-7 cells treated IKKVII (1 μM) or GTCpFE (50 μM) for 2 hours, followed by TNFα treatment for 15 minutes. The different letters above bars indicate significant difference between treatments (*P* < 0.001). **d** Whole cell extracts of cells treated as in (C) were prepared and NFκB signaling proteins were examined by western blotting. Representative western blots from three independent experiments are shown. β-actin served as a loading control. **e**-**f** TNFα-induced expression of ICAM1, CCL2 and TNF in (E) BT474 or (F) MDA-MB-231 cells was measured after pretreatment with varying concentrations of GTCpFE

**Fig. 4:**
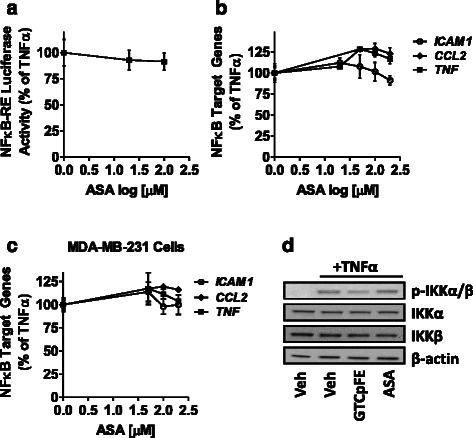
ASA cannot inhibit NFκB activity in breast cancer cells. **a** NFκB-RE activity or **b** NFκB target gene expression (ICAM1, CCL2 and TNF) was measured in MCF-7 cells treated with different concentrations of ASA. **c** RT-QPCR for NFκB target gene expression was measured in MDA-MB-231 cells treated with increasing concentrations of ASA. TNFα was used to activate NFκB, and ASA response is plotted as % of TNFα alone. **d** ASA cannot inhibit TNFα induced phosphorylation of IKKs. MCF-7 cells were pretreated with 50 μM GTCpFE or ASA for 2 hours followed by TNFα for 15 minutes. Phosphorylated and total IKK levels were examined by western blotting. β-actin served as a loading control

Dose response studies were conducted in MCF-7 cells and GTCpFE was found to inhibit both NFκB-RE activity and expression of NFκB target genes, such as ICAM1, CCL2 and TNF, with a calculated IC_50_ value of ~20 μM (Fig. 3a, b). In addition, GTCpFE was found to inhibit NFκB activity in other breast cancer cell lines, such as BT474 and MDA-MB-231 (Fig. 3e, f), and in response to other cytokines, including IL-1β (Additional file 3: Figure S2). In contrast, ASA alone had no effect on NFκB-RE activity and expression of target genes in breast cancer cells even at doses as high as 200 μM (Fig. 4a-c).

The canonical NFκB pathway consists of p65 and p50 transcription factors, which are held in the cytoplasm by an inhibitor protein, IκBα. Upon stimulation by inflammatory cytokines (such as TNFα, IL-1β) or other factors, the IκB kinase (IKK) complex is activated, leading to phosphorylation and degradation of IκBα. As a result, p65/p50 factors are liberated and can translocate to the nucleus, where they bind to DNA. To determine where in this pathway GTCpFE may be acting, we first examined DNA binding activity of the NFκB family member, p65 (RelA). GTCpFE inhibits p65 DNA binding by ~50 %, which is comparable in this assay to the known IKKα/β inhibitor, IKKVII (Fig. 3c). We next examined upstream components in the NFκB signaling pathway and found that IKKα/β phosphorylation, IκBα phosphorylation and degradation, and p65 phosphorylation were impaired by GTCpFE (Fig. 3d). These data indicate that GTCpFE, but not ASA (Fig. 4d), is capable of inhibiting NFκB activity in breast cancer cells, by blocking IKKα/β phosphorylation and subsequent activation of the p65 transcription factor. Thus, GTCpFE represents a significant improvement compared to ASA on NFκB pathway inhibition.

### GTCpFE inhibits breast cancer stem cell properties

**Fig. 5:**
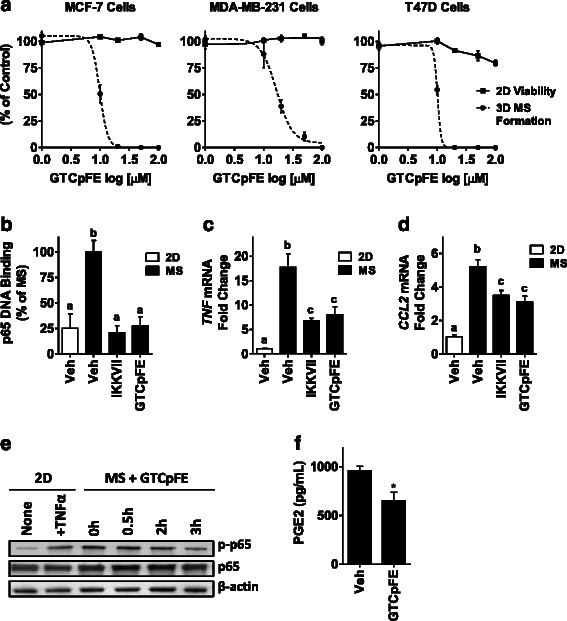
The effects of GTCpFE in MS culture of breast cancer cells. **a** MS formation and cell viability from indicated cell lines was measured after treatment with varying concentrations of GTCpFE. The effect of GTCpFE in both assays is plotted as % of DMSO vehicle control. **b** p65 DNA binding activity was measured in conventional adherent 2D culture of MCF-7 cells or MS culture with or without inhibitors (IKKVII 1 μM or GTCpFE 50 μM) added for the last 3 hours. **c**-**d** TNF (C) and CCL2 (D) expression after 6 hours was measured in treatment groups described in (B). The different letters (a, b, c) above bars indicate significant difference between treatments, *P* < 0.05. **e** Total and p-p65 levels are measured in 2D vs MS culture treated with 50 μM GTCpFE. **f** PGE2 levels in the media of MDA-MB-231 MS was measured upon treatment with 20 μM GTCpFE for 24 hours, * *P* < 0.05

Because the breast CSC phenotype has been shown to dependent on both COX2/PGE2 [25–28] and NFκB activity [22–24], we next explored whether GTCpFE could affect formation of mammospheres (MS), which are enriched for cells with the stem-like properties of self-renewal and anchorage-independent growth [29, 30]. GTCpFE prevented MS formation in a dose-dependent manner in all breast cancer cell lines examined (Fig. 5a). Importantly, MS inhibition occurred at doses that do not affect cell viability in standard adherent monolayer cultures (Fig. 5a). To determine if GTCpFE can affect the NFκB pathway or PGE2 production in breast CSCs, MS were allowed to form over 7 days and inhibitors were added for the last 3–24 hours of culture. MS displayed elevated levels of p65 DNA binding, NFκB target gene expression, and p65 phosphorylation compared to untreated breast cancer cells cultured in standard monolayer (2D) conditions. All of these endpoints were attenuated by GTCpFE (Fig. 5b-e). Also, PGE2 production is reduced in MS treated with GTCpFE, confirming the expected ASA-like activity (Fig. 5f). Together, these findings suggest that GTCpFE can block MS formation by inhibiting both the NFκB pathway and PGE2 production.

**Fig. 6:**
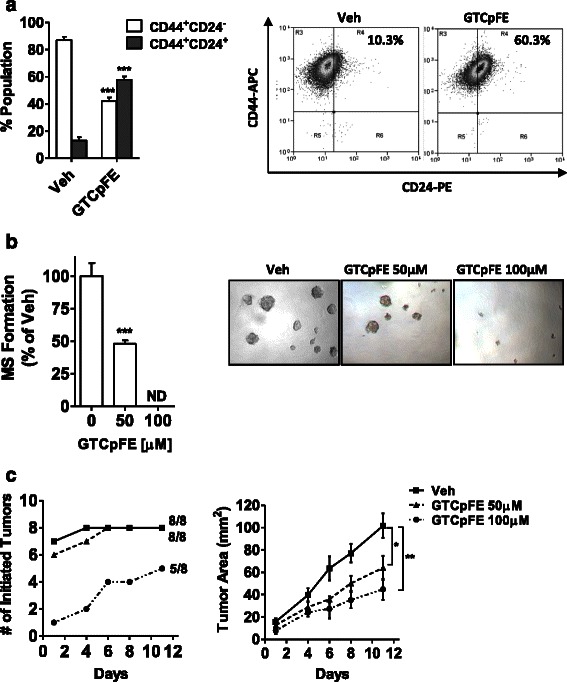
GTCpFE pre-treatment reduces the CD44^+^CD24^−^ population, MS growth, and tumor initiation capacity. **a** The CD44^+^CD24^−^ population was determined by FACS analysis of MDA-MB-231 cells treated with 50 μM GTCpFE for 72 hours. Quantitation of each population percentage (left) and representative scatter plots from FACS (right) are shown. *** *P* < 0.001. **b** MS formation was measured following pretreatment of MDA-MB-231 cells with 50 or 100 μM GTCpFE for 72 hours. GTCpFE is then withdrawn prior to seeding in MS culture. MS growth inhibition was plotted as % of DMSO vehicle control (left) and representative pictures of MS growth are shown (10×, right). *** *P* < 0.001; ND, none detected. **c** The number of xenograft tumors initiated over time (left panel) and their growth rates (right panel) was determined from GTCpFE or vehicle pre-treated MDA-MB-231 cells. * *P* < 0.05, ** *P* < 0.01

We next conducted follow-up studies to confirm that GTCpFE is in fact targeting breast CSCs. For these studies, MDA-MD-231 cells were selected since they have previously been shown to contain a higher percentage of CSCs [57, 58], given their mesenchymal character [59]. MDA-MB-231 cells were pre-treated for 72 hours with GTCpFE, followed by washing and measurement of three well-established CSC endpoints: CD44^+^CD24^−^ cell surface marker expression, MS formation, and xenograft tumor initiation. GTCpFE pre-treatment resulted in a significant depletion of the CD44^+^CD24^−^ population (Fig. 6a). Similarly, consistent with the depletion of CSCs, we observe that GTCpFE pre-treated cells are functionally less capable of MS formation, even in the absence of continued GTCpFE treatment (Fig. 6b).

The “gold standard” for assaying anti-CSC properties is *in vivo* tumorigenicity, wherein the ability of drug-treated cells to initiate or seed a xenograft tumor is examined [60, 61]. Because CSCs are the population present in each cell line capable of tumorigenicity, a drug that attenuates this population, is reflected in reduced tumor initiation capacity or incidence. MDA-MB-231 cells were treated with GTCpFE (50 μM or 100 μM) for 72 hours, followed by washing and injection into the mammary fat pad of female athymic nude mice. GTCpFE at 100 μM decreased the overall number of tumors that formed (Fig. 6c, left). Furthermore, of the tumors that did form from GTCpFE pre-treated cells, latency was increased (Fig. 6c, left), and the growth rate was significantly reduced (Fig. 6c, right). Altogether, these data confirm that the novel anti-inflammatory agent, GTCpFE, is also a potent anti-CSC agent.

### Structural components of GTCpFE necessary for anti-NFκB and anti-CSC activity

**Fig. 7:**
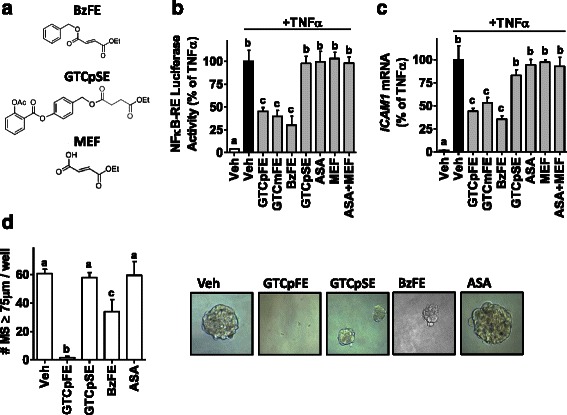
The fumarate moiety of GTCpFE is required for the NFκB inhibition in breast cancer cells. **a** Structural analogs of GTCpFE are indicated. **b** NFκB-RE activity was measured in MCF-7 cells following treatment with TNFα and the analogs shown in (A) 20 μM each. **c** RT-QPCR for ICAM1 gene expression was measured in MCF-7 cells treated as in (B). **d** MS formation of MCF-7 cells treated with 20 μM GTCpFE, ASA, BzFE, and GTCpSE demonstrate attenuated MS growth. Quantitation of MS growth (left panel) and representative pictures (20×) of MS (right panel) are shown. The different letters above bars (a, b, c) indicate significant difference between treatments, *P* < 0.05

Since GTCpFE inhibits the NFκB pathway and PGE2 production, and is capable of attenuating breast CSCs without non-specific toxicity, we next examined what components of its structure contribute to its activity. A series of compounds with truncated or inactivated moieties were tested (Fig. 7a). For anti-NFκB activity, the fumarate group appears to be essential since GTCmFE, the *meta* isomer of GTCpFE, retains its inhibitory function (Fig. 7b, c). Also, BzFE, which lacks the ASA moiety but retains the fumarate, inhibits the NFκB pathway in a similar manner to GTCpFE (Fig. 7b, c). Furthermore, GTCpSE, which consists of ASA linked to succinate, a structural analog of fumarate that lacks the reactive double bond, is not capable of inhibiting the NFκB pathway (Fig. 7b, c). These findings indicate that the fumarate component of GTCpFE is necessary to elicit the observed inhibition of the NFκB pathway in breast cancer cell lines. Interestingly, when we tested the hydrolysis products of GTCpFE – ASA and monoethyl fumarate (MEF), either alone or in combination – no effect was observed (Fig. 7b, c). This implies that fumarate within an intact prodrug is required for the anti-inflammatory activity of GTCpFE on the NFκB pathway.

For anti-CSC activity of GTCpFE, both the fumarate and the ASA moieties are required. An ASA prodrug with inactivated fumarate, GTCpSE, has no effect on MS growth (Fig. 7d), suggesting the fumarate is required. However, the fumarate alone is not sufficient, because the analog lacking the ASA moiety, BzFE, has little effect on MS formation (Fig. 7d). We also tested IKKVII for its effect on the CD44^+^CD24^−^ immunophenotype Additional file 4: Figure S3), and found that it modestly attenuates the CD44^+^CD24^−^ population compared to GTCpFE (Figure 6a). This suggests that NFκB inhibition, whether by fumarates or IKKVII, is only partially effective on CSCs. Similarly, the ASA moiety alone is not sufficient, because ASA has no effect. This in addition to GTCpSE data indicates that simple COX inhibition is not sufficient either. Altogether, these findings suggest that both the anti-NFκB and anti-COX activity of GTCpFE are required for its superior anti-CSC effect.

## Discussion

In this study, we have demonstrated that ASA prodrugs with ancillary pharmacophores can be effectively used to inhibit NFκB activity in breast cancer cells, whereas ASA itself was ineffective at much higher concentrations. While we hypothesized that prodrug isomerism (*para* vs *meta*) would be important [47], our data indicate that in fact pharmacophore reactivity is the main driver of biological activity. For instance, we find several highly reactivity pharmacophores, but they also display inherent cytotoxicity. Instead, incorporation of the fumarate pharmacophore, as in GTCpFE, proved to be the optimal moiety in balancing potent anti-NFκB activity versus no concomitant cytotoxicity. We find GTCpFE to effectively inhibit NFκB activation in a breast cancer subtype-independent manner demonstrated in multiple breast cancer cell lines and the intrinsic NFκB activity essential for CSCs.

Interestingly, the fumarate alone is not sufficient to inhibit NFκB but that its presence within the intact prodrug is required. The activity of GTCpFE was not seen in an analogue, GTCpSE, identical except for the presence of the fumarate structural element, and therefore, is ascribed to the fumarate pharmacophore, designed to enhance anti-NFκB activity. However, the finding that MEF, either alone or in combination with ASA had no effect suggests that fumarate alone is not sufficient to inhibit NFκB but required as part of the prodrug. Likewise, the simple ASA prodrug approach is not sufficient to inhibit NFκB. Innumerable studies have shown that in cell culture, ASA itself has very low potency, whereas cell-permeable ester prodrugs are dramatically more potent as anti-inflammatory or anti-cancer agents. Accordingly, GTCpSE was designed as an ASA ester prodrug, identical to GTCpFE in all aspects but the fumarate, yet it shows no activity against the NFκB pathway. Together these observations demonstrate the importance of the fumarate pharmacophore and show that the anti-NFκB activity of GTCpFE goes beyond that of a simple ASA ester prodrug.

Use of fumarates as anti-inflammatory agents is not unprecedented; dimethyl fumarate (Tecfidera®) is an approved anti-inflammatory drug that has been shown to inhibit NFκB signaling in a variety of cell lines [49–53]. The mechanism by which dimethyl fumarate inhibits the NFκB pathway is unclear but does not appear to involve the upstream IKKs. Rather, nuclear entry and phosphorylation of NFκB transcription factors is attenuated and other kinases such as MSK-1 appear to be involved [50, 53]. In contrast, our studies demonstrate that GTCpFE inhibits IKKα/β activity and subsequent activation of the p65 transcription factor. This may be a new mode of action for this particular ASA prodrug that extends beyond that of the parent drug.

Our findings suggest that GTCpFE may also be a promising, clinically relevant anti-inflammatory molecule for eradication of breast CSCs by exploiting CSC’s reliance on multiple inflammatory pathways [22–26]. GTCpFE, at concentrations where MS formation is completely abrogated, showed little to no effect on viability of adherent parental cells. In addition, the promising *in vitro* anti-CSC properties of GTCpFE translated to attenuated tumorigenicity of MDA-MB-231 xenografts compatible with the diminution of the CSC population by GTCpFE. Interestingly, inhibition of CSCs requires both the anti-NFκB activity and retention of ASA-like activity on COX-PGE2 axis. Testing whether these findings on the anti-CSC activity of GTCpFE in a xenograft model also translate in an immunocompetent transgenic mouse model of breast cancer would be of great interest and subject to future studies.

Targeting breast CSCs, which are at the apex of the tumor hierarchy, is increasingly recognized as fundamental to effective anti-cancer therapy. Breast CSCs, also referred to as tumor-initiating cells, are highly tumorigenic, can evade anoikis, and are capable of self-renewal and asymmetrical division; and thereby can reconstitute intratumoral heterogeneity [29–31]. Breast CSCs were shown to be resistant to treatment with chemotherapeutics and ionizing radiation [33, 34]. They also display epithelial-mesenchymal transition features, and thus are thought to mediate tumor metastasis and tumor recurrence [36, 37]. These properties of breast CSCs negatively impact clinical outcome, highlighting the need for new therapeutic strategies to target CSCs. Currently, standard therapeutic drugs are seen as ineffective in killing CSCs and no specific CSC agents have been approved.

## Conclusion

Based on our studies, we conclude that the fumarate-based ASA prodrug, GTCpFE, described herein, is a prototype for developing new anti-inflammatory and anti-CSC class of drugs with the potential to impact aggressive breast cancers.

## Additional files

Additional file 1: **Supplemental Methods.** Synthetic procedures and characterization of chemicals used are indicated. (PDF 762 kb)
Additional file 2: Figure S1. Synthesis of ASA prodrugs. Chemical structures and synthetic schemes are indicated and described in Additional file 1: Supplemental Methods. (PPTX 133 kb)
Additional file 3: Figure S2. GTCpFE inhibits cytokine-induced NFκB target gene expression in breast cancer cells. MCF-7 cells were pretreated for 2 hours with increasing concentrations of GTCpFE followed by treatment with IL-1β (10 ng/ml) for another 2 hours. Expression of NFκB target genes, ICAM1 and CCL2 was measured by RT-QPCR. Drug inhibitory activity is plotted as % of IL-1β alone. (PPTX 72 kb)
Additional file 4: Figure S3. The effect of NFκB inhibitor, IKKVII, on the CD44^+^CD24^−^ population. The CD44^+^CD24^−^ population percentage was determined by FACS analysis of MDA-MB-231 cells treated with 2.5 μM IKKVII for 72 hours. * *P* < 0.05. (PPTX 48 kb)

## Abbreviations

ASA: acetylsalicylic acid, aspirin
COX: cyclooxygenase
CSC: cancer stem cell
MS: mammosphere
NFκB: nuclear factor κB
NSAID: non-steroidal anti-inflammatory drug
PG: prostaglandin
