# Supplementary material for: A novel aspirin prodrug inhibits NFκB activity and breast cancer stem cell properties
Source: BMC Cancer. 2015 Nov 4;15:845. doi: 10.1186/s12885-015-1868-7 (PMC4632459; doi:10.1186/s12885-015-1868-7)

Supplemental Methods

General procedure. Under a positive pressure of argon and free of moisture, a solution of either 3- or 4-hydroxymethylphenol ester of 2-acetyloxybenzoic acid[1,2] (100 mg, 0.349 mmol), 4-dimethylaminopyridine (4 mg, 0.033 mmol) and triethylamine (53 mg, 0.523 mmol) in anhydrous tetrahydrofuran (5 mL) was cooled to 0 ºC, and a solution of the appropriate acyl chloride (0.419 mmol) in anhydrous tetrahydrofuran (5 mL) was added dropwise over a period of 10 min. The resulting solution was stirred at 0-5 ºC for 2-4 hours. The reaction mixture was diluted with ethyl acetate (100 mL) and the diluted solution was washed with brine (50 mL x 3), dried over sodium sulfate, evaporated to dryness, and the residue was purified by column chromatography on silica gel.

GTCpFE. 4-Hydroxymethylphenol ester of 2-acetyloxybenzoic acid and ethyl fumaroyl chloride were used. Purification using AcOEt/hexane (20:80) as eluent afforded 98 mg (68%) of the desired product as a white solid. ^{1}H NMR ($CDCl_3$): δ 1.29 (t, 3 H, 3J = 7.0 Hz, CH_3); 2.31 (s, 3 H, CH_3); 4.26 (q, 2 H, 3J = 7.0 Hz, $COOCH_2$); 5.25 (s, 2 H, OCH_2); 6.89 (s, 2 H, HC=CH); 7.19 (m, 3 H, Ar); 7.42 (m, 3 H, Ar); 7.65 (m, 1 H, Ar); 8.22 (dd, 1 H, 3J = 7.8, 4J = 1.2 Hz, Ar). ^{13}C NMR ($CDCl_3$) δ: 169.70, 164.86, 164.75, 162.89, 151.28, 150.69, 134.76, 134.32, 133.26, 133.16, 132.25, 129.81, 126.26, 124.11, 122.47, 122.04, 66.40, 61.43, 21.05, 14.15. HRMS ($M+NH_4^+$) calc'd: 430.1496; observed: 430.1477.

GTCmFE. 3-Hydroxymethylphenol ester of 2-acetyloxybenzoic acid and ethyl fumaroyl chloride were used. Purification using AcOEt/hexane (20:80) as eluent afforded 135 mg (94%) of the desired product as a white solid. ^{1}H NMR ($CDCl_3$): δ 1.30 (t, 3 H, 3J = 7.1 Hz, CH_3); 2.30 (s, 3 H, CH_3); 4.24 (q, 2 H, 3J = 7.1 Hz, $COOCH_2$); 5.25 (s, 2 H, OCH_2); 6.89 (s, 2 H, HC=CH); 7.17 (d, 2 H, 3J = 8.0 Hz, Ar); 7.21 (s, 1 H, Ar), 7.27 (d, 2 H, 3J = 7.7 Hz, Ar); 7.40

(m, 2 H, Ar); 7.63 (m, 1 H, Ar); 8.25 (dd, 1 H, 3J = 7.8, 4J = 1.1 Hz, Ar). ^{13}C NMR ($CDCl_3$) δ: 169.63, 164.76, 164.63, 162.80, 151.22, 150.71, 137.13, 134.69, 134.32, 133.99, 132.18, 129.83, 126.19, 125.77, 124.05, 122.37, 121.82, 121.41, 66.23, 61.36, 20.97, 14.07. HRMS ($M+H^+$) calc'd: 413.1231; observed: 413.1214.

GTCpSE. 4-Hydroxymethylphenol ester of 2-acetyloxybenzoic acid and ethyl 4-chloro-4-oxobutyrate were used. Purification using AcOEt/hexane (25:75) as eluent afforded 106 mg (73%) of the desired product as clear oil. ^{1}H NMR ($CDCl_3$): δ 1.23 (t, 3 H, J = 7.1 Hz, CH_3); 2.30 (s, 3 H, CH_3); 2.64 (AB d, 2 H, 3J = 5.3 Hz, CH_2COO); 2.68 (AB d, 2 H, 3J = 5.3 Hz, CH_2COO); 4.33 (q, 2 H, 3J = 7.1 Hz, $COOCH_2$); 5.14 (s, 2 H, OCH_2); 7.17 (d, 3 H, 3J = 8.3 Hz, Ar); 7.38 (m, 3 H, Ar); 7.63 (t, 1 H, 3J = 7.1 Hz, Ar); 8.20 (d, 2 H, 3J = 7.1 Hz, Ar). ^{13}C NMR ($CDCl_3$) δ: 172.17, 172.07, 169.64, 162.85, 151.18, 150.41, 134.66, 133.75, 132.17, 129.57, 126.18, 124.02, 122.42, 121.84, 65.77, 60.70, 29.15, 29.10, 20.96, 14.14. HRMS ($M+H^+$) calc'd 415.1387; observed: 415.1392.

BzFE. This compound was prepared similarly to the reported procedure.[3] To a solution of monoethyl fumarate (2.88 g, 20.0 mmol) in anhydrous dichloromethane (20 mL) benzyl alcohol (6.20 mL, 6.49 g, 60 mmol) and 4-DMAP (2.4 g, 20 mmol) were added. The mixture was chilled at 0 ^{o}C in ice-water bath, followed by addition of DCC (4.5 g, 22 mmol). The mixture was stirred for 5 minutes, then the cold bath was removed, and the reaction mixture was stirred for further 36 hours. The reaction mixture was then filtered, the filtrate was washed with dichloromethane. Combined organic solutions were washed with water (10 mL), dried over Na_2SO_4, concentrated under reduced pressure, and purified by column chromatography on silica gel (hexane-ethyl acetate 20:1) to afford 2.75 g (59%) of product. ^{1}H NMR ($CDCl_3$): δ 1.31 (t, 3 H, 3J = 7.2 Hz, CH_3); 4.25 (q, 2 H, 3J = 7.2 Hz, OCH_2); 5.23 (s, 2 H, OCH_2); 6.89 (s, 2 H,

HC=CH); 7.37 (m, 5 H, Ar). ^{13}C NMR ($CDCl_3$) δ: 164.85, 164.77, 135.24, 134.10, 133.24, 128.64, 128.49, 128.32, 67.05, 61.35, 14.08.

References

1 Dunlap T, Abdul-Hay SO, Chandrasena RE, Hagos GK, Sinha V, Wang Z et al. Nitrates and NO-NSAIDs in cancer chemoprevention and therapy: in vitro evidence querying the NO donor functionality. Nitric Oxide. 2008; **19**: 115-124.

2 Dunlap T, Piyankarage SC, Wijewickrama GT, Abdul-Hay S, Vanni M, Litosh V et al. Quinone-induced activation of Keap1/Nrf2 signaling by aspirin prodrugs masquerading as nitric oxide. Chem Res Toxicol. 2012; **25**: 2725-2736.

3 Xue C-B, Decicco CP, He X. Cyclic hydroxamic acids as metalloproteinase inhibitors. PCT Int Appl. 1999; WO 9965867 A9965861 19991223.

Spectral characterizations for ASA prodrugs.

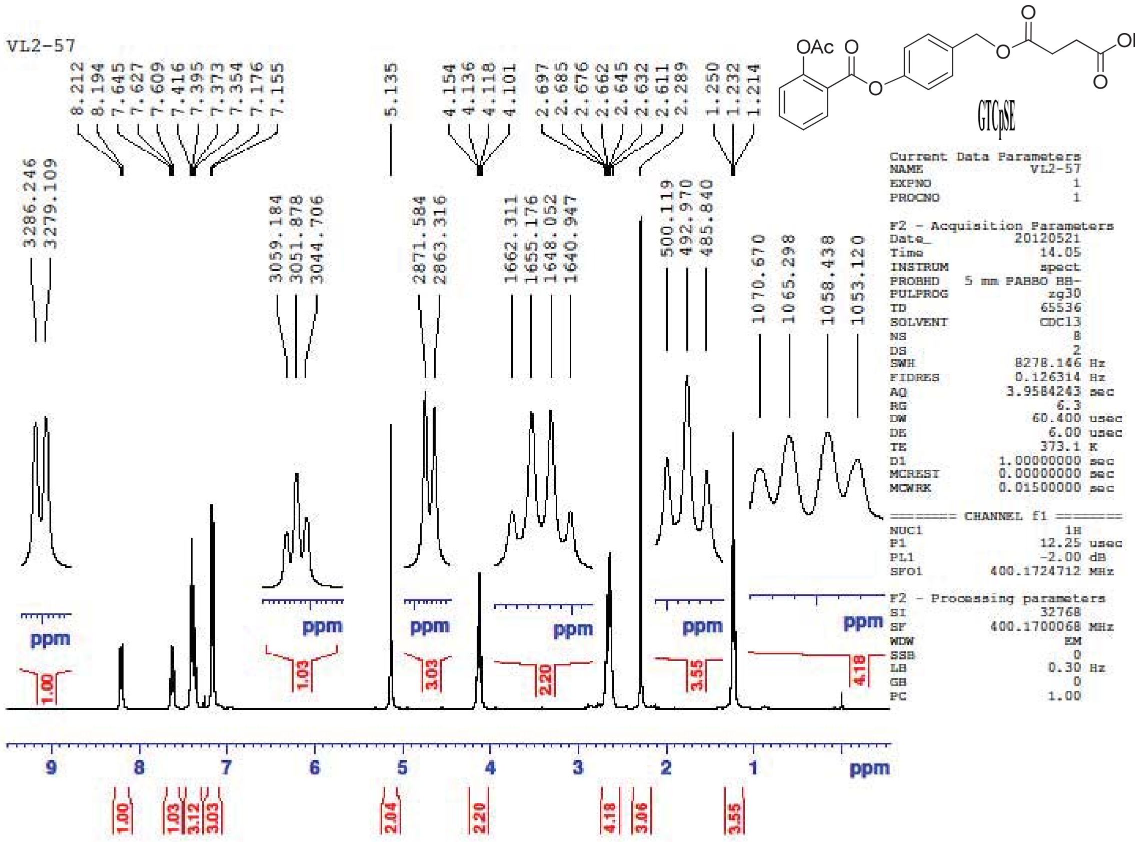
VL2-57
8.212 8.194 7.645 7.627 7.609 7.416 7.395 7.373 7.354 7.176 7.155
5.135
4.154 4.136 4.118 4.101
2.697 2.685 2.676 2.662 2.645 2.632 2.611 2.289
1.250 1.232 1.214
3286.246 3279.109
3059.184 3051.878 3044.706
2871.584 2863.316
1662.311 1655.176 1648.052 1640.947
500.119 492.970 485.840
1070.670 1065.298 1058.438 1053.120
OAc
OEt
GTCpSE
Current Data Parameters
NAME VL2-57
EXPNO 1
PROCNO 1
F2 - Acquisition Parameters
Date_ 20120521
Time 14.05
INSTRUM spect
PROBHD 5 mm PABBO BB-
PULPROG zg30
TD 65536
SOLVENT CDCl3
NS 8
DS 2
SWH 8278.146 Hz
FIDRES 0.126314 Hz
AQ 3.9584243 sec
RG 6.3
DW 60.400 usec
DE 6.00 usec
TE 373.1 K
D1 1.00000000 sec
MCREST 0.00000000 sec
MCWRK 0.01500000 sec
======== CHANNEL f1 ========
NUC1 1H
P1 12.25 usec
PL1 -2.00 dB
SFO1 400.1724712 MHz
F2 - Processing parameters
SI 32768
SF 400.1700068 MHz
WDW EM
SSB 0
LB 0.30 Hz
GB 0
PC 1.00
ppm
1.00 1.03 3.03 2.20 3.55 4.18
9 8 7 6 5 4 3 2 1 ppm
1.00 1.03 3.12 3.03 2.04 2.20 4.18 3.06 3.55

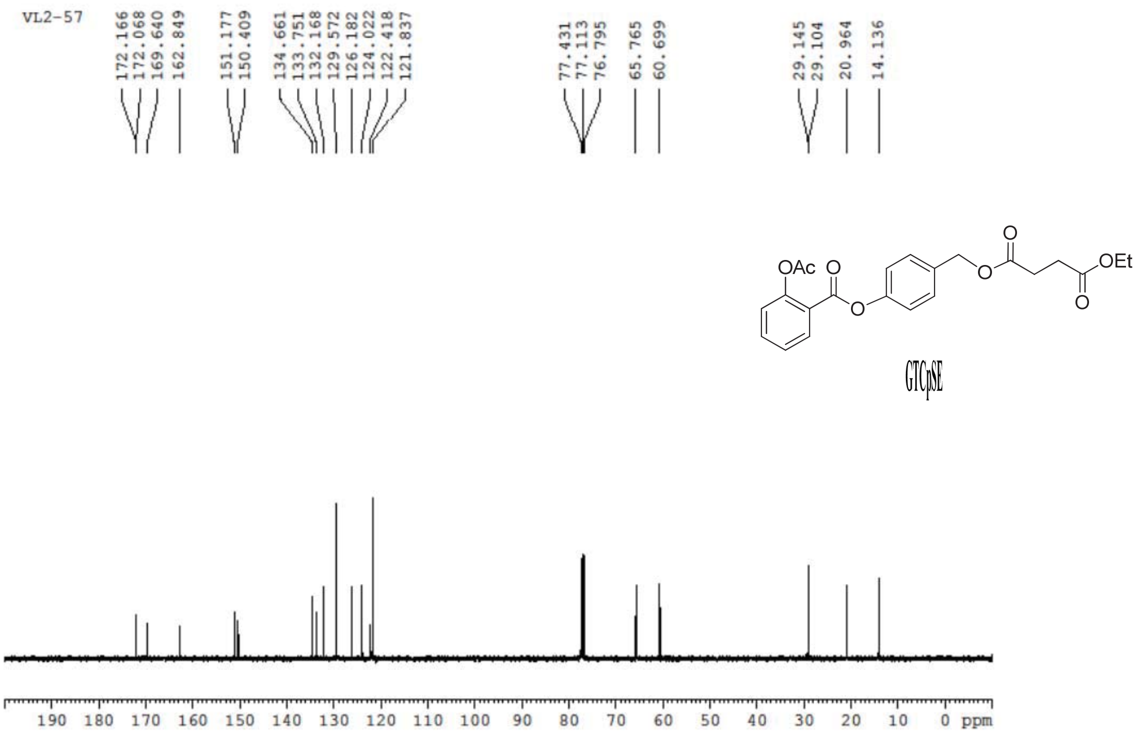
VL2-57
172.166 172.068 169.640 162.849 151.177 150.409 134.661 133.751 132.168 129.572 126.182 124.022 122.418 121.837
77.431 77.113 76.795 65.765 60.699
29.145 29.104 20.964 14.136
OAc
OEt
GTCpSE
190 180 170 160 150 140 130 120 110 100 90 80 70 60 50 40 30 20 10 0 ppm

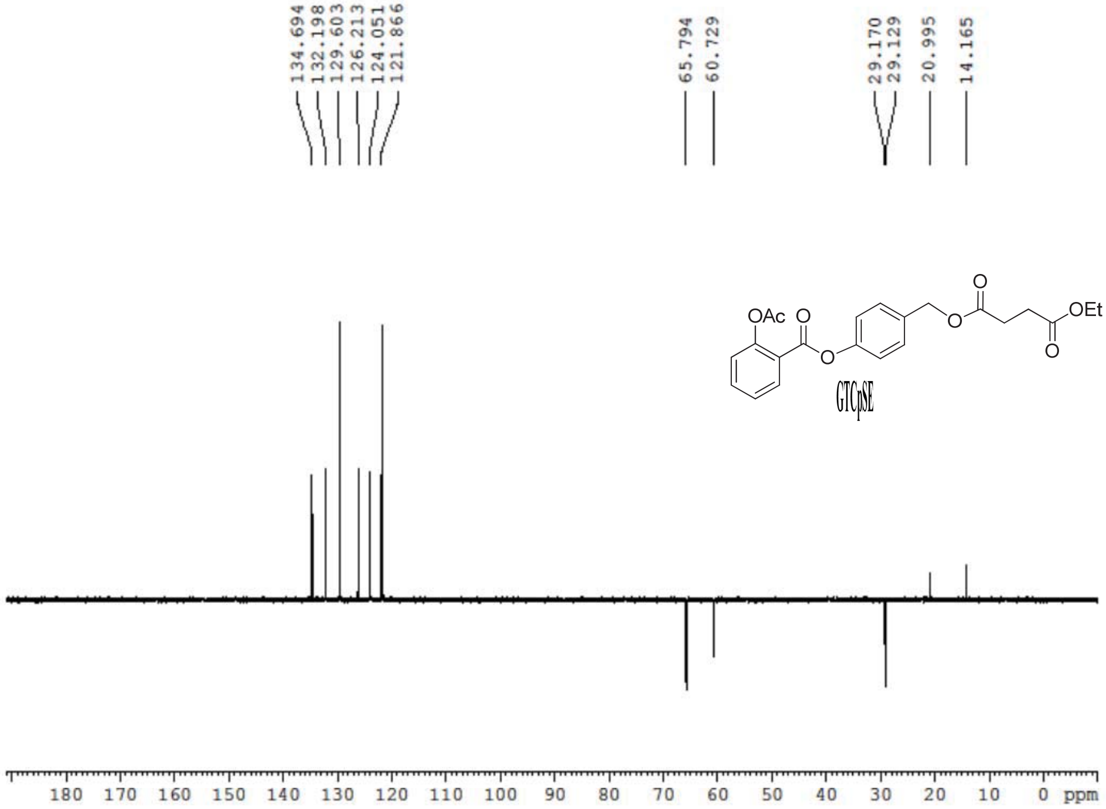

Shimadzu IT-TOF Accurate Mass Report

Sample Name	: C	RRC Sample Number: C
Data File Name	: 20120524_rrc_log__C_5.lcd	
Data Acquired	: 5/24/2012	

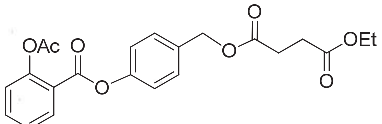

GTCpSE

Mass Spectrum

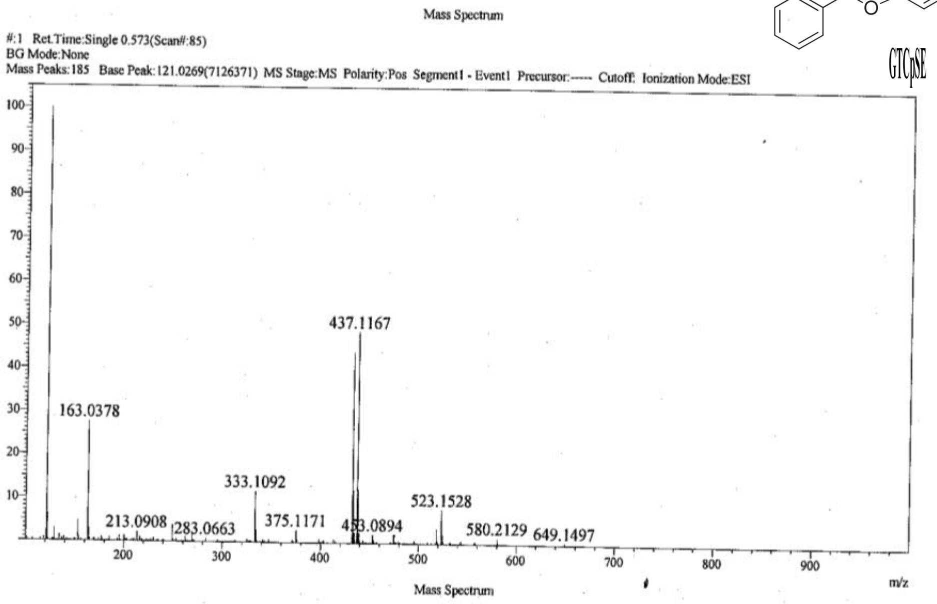

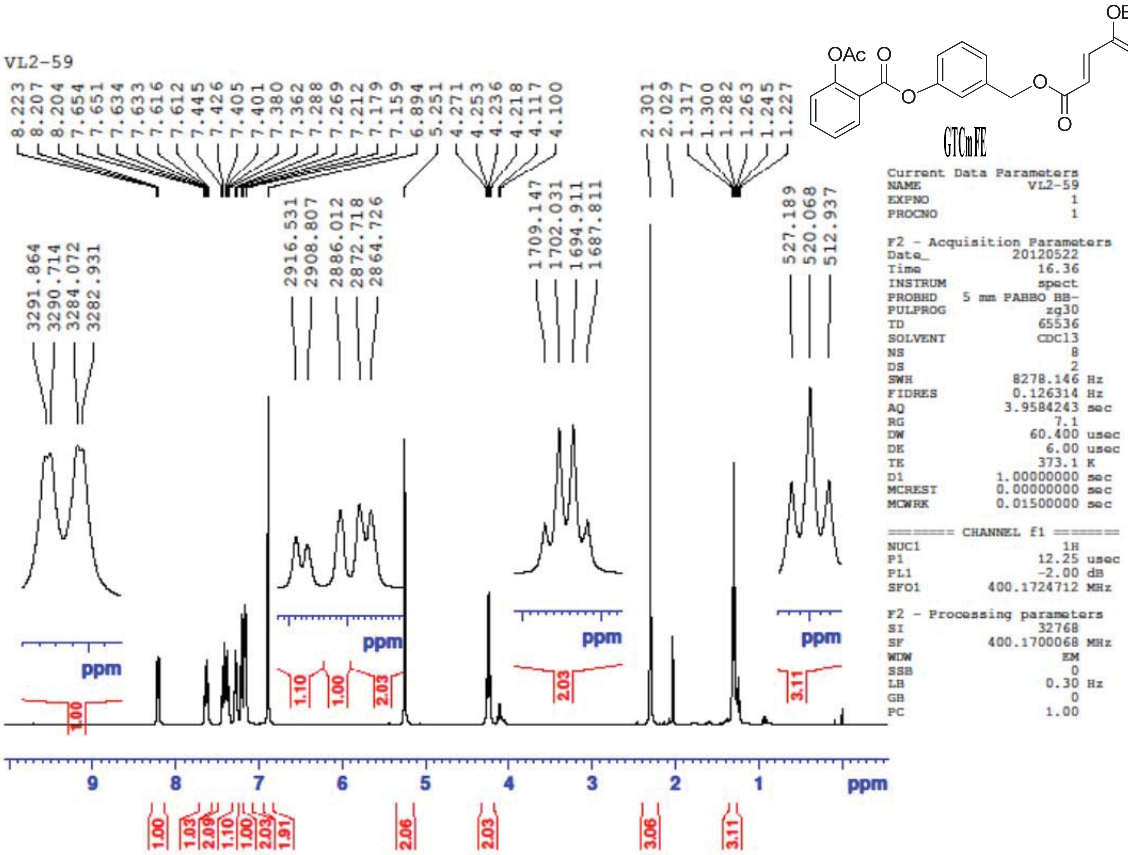
VL2-59
8.223 8.207 8.204 7.654 7.651 7.634 7.633 7.616 7.612 7.445 7.426 7.405 7.401 7.380 7.362 7.288 7.269 7.212 7.179 7.159 6.894 5.251 4.271 4.253 4.236 4.218 4.117 4.100 2.301 2.029 1.317 1.300 1.282 1.263 1.245 1.227
3291.864 3290.714 3284.072 3282.931
2916.531 2908.807 2886.012 2872.718 2864.726
1709.147 1702.031 1694.911 1687.811
527.189 520.068 512.937
OAc
OEt
GTCmFE
Current Data Parameters
NAME VL2-59
EXPNO 1
PROCNO 1
F2 - Acquisition Parameters
Date_ 20120522
Time 16.36
INSTRUM spect
PROBHD 5 mm PABBO BB-
PULPROG zg30
TD 65536
SOLVENT CDCl3
NS 8
DS 2
SWH 8278.146 Hz
FIDRES 0.126314 Hz
AQ 3.9584243 sec
RG 7.1
DW 60.400 usec
DE 6.00 usec
TE 373.1 K
D1 1.00000000 sec
MCREST 0.00000000 sec
MCWRK 0.01500000 sec
======= CHANNEL f1 =======
NUC1 1H
P1 12.25 usec
PL1 -2.00 dB
SFO1 400.1724712 MHz
F2 - Processing parameters
SI 32768
SF 400.1700068 MHz
WDW EM
SSB 0
LB 0.30 Hz
GB 0
PC 1.00
ppm
1.00
1.10 1.00 2.03
2.03
3.11
9 8 7 6 5 4 3 2 1 ppm
1.00 1.03 2.09 1.10 1.00 2.03 1.91 2.06 2.03 3.06 3.11

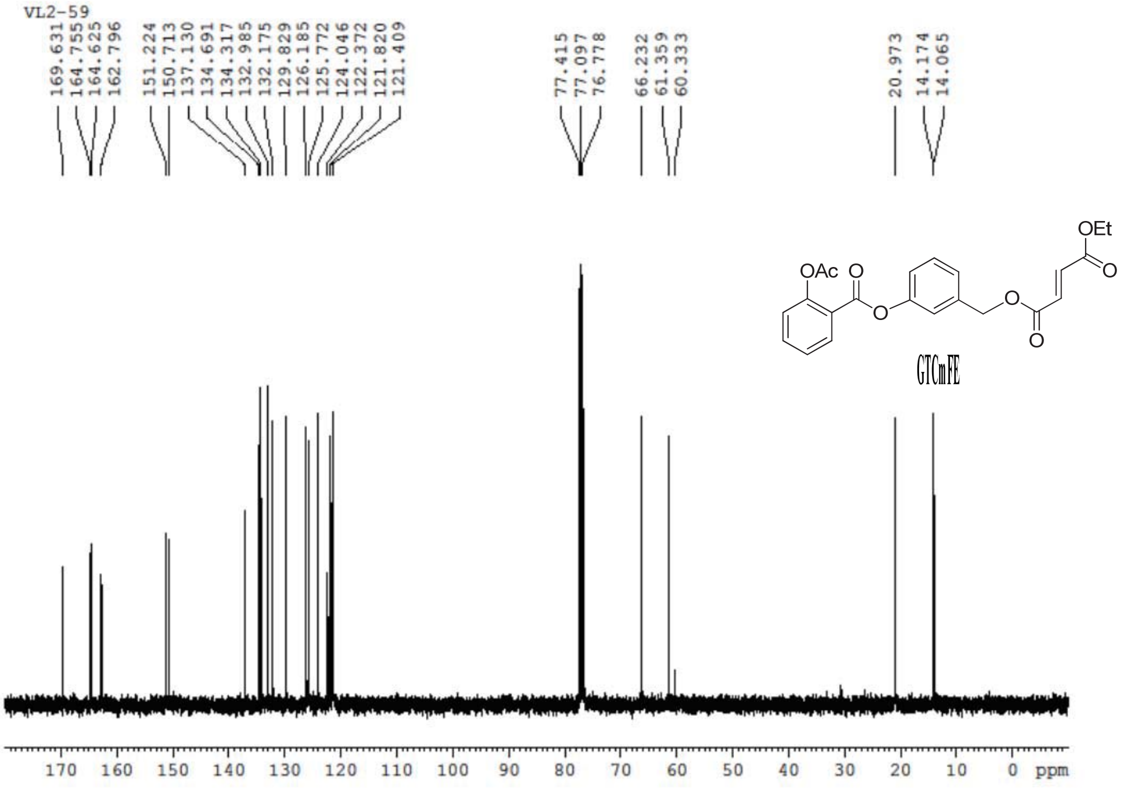
VL2-59
169.631 164.755 164.625 162.796 151.224 150.713 137.130 134.691 134.317 132.985 132.175 129.829 126.185 125.772 124.046 122.372 121.820 121.409 77.415 77.097 76.778 66.232 61.359 60.333 20.973 14.174 14.065
OAc
OEt
GTCmFE
170 160 150 140 130 120 110 100 90 80 70 60 50 40 30 20 10 0 ppm

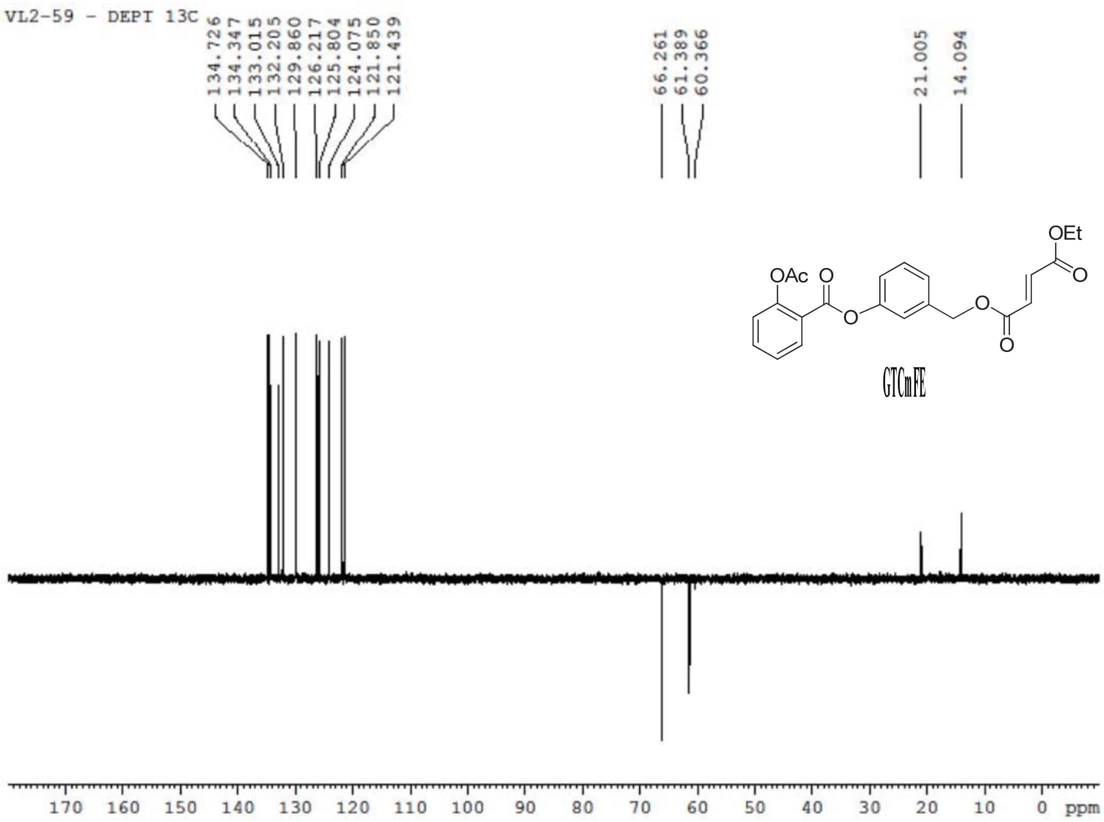

Shimadzu IT-TOF Accurate Mass Report

RRC Sample Number: A

Sample Name : A
Data File Name : 20120524_rrc_log__A_3.lcd
Data Acquired : 5/24/2012

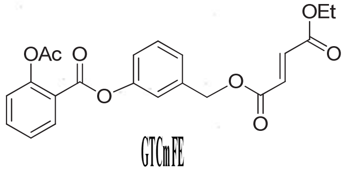

Mass Spectrum

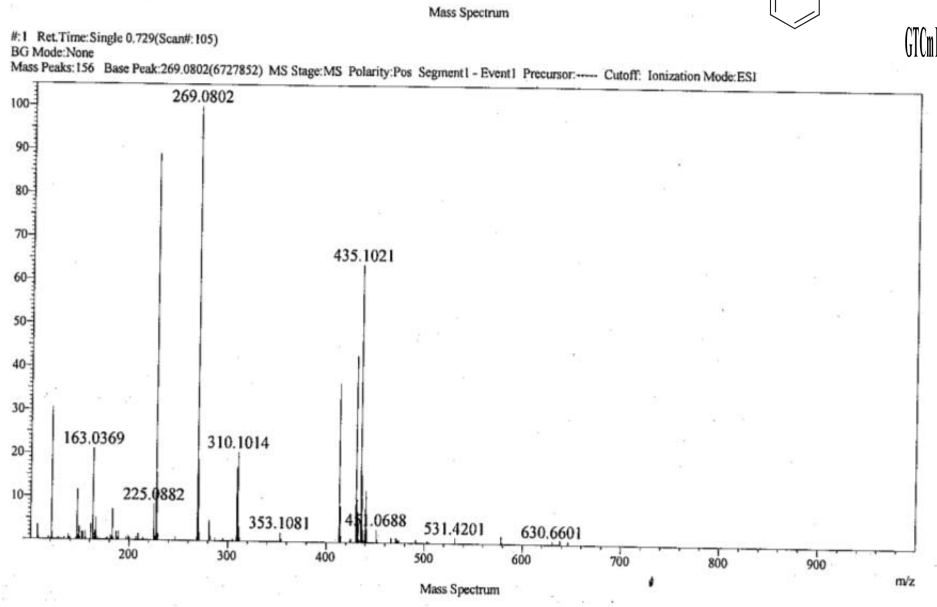

Supplement: Additional file 1: — Supplemental Methods. Synthetic procedures and characterization of chemicals used are indicated. (PDF 762 kb) [file 12885_2015_1868_MOESM1_ESM.pdf]
