# Supplementary figures and images for: A novel aspirin prodrug inhibits NFκB activity and breast cancer stem cell properties
Source: BMC Cancer. 2015 Nov 4;15:845. doi: 10.1186/s12885-015-1868-7 (PMC4632459; doi:10.1186/s12885-015-1868-7)

## Slide 1
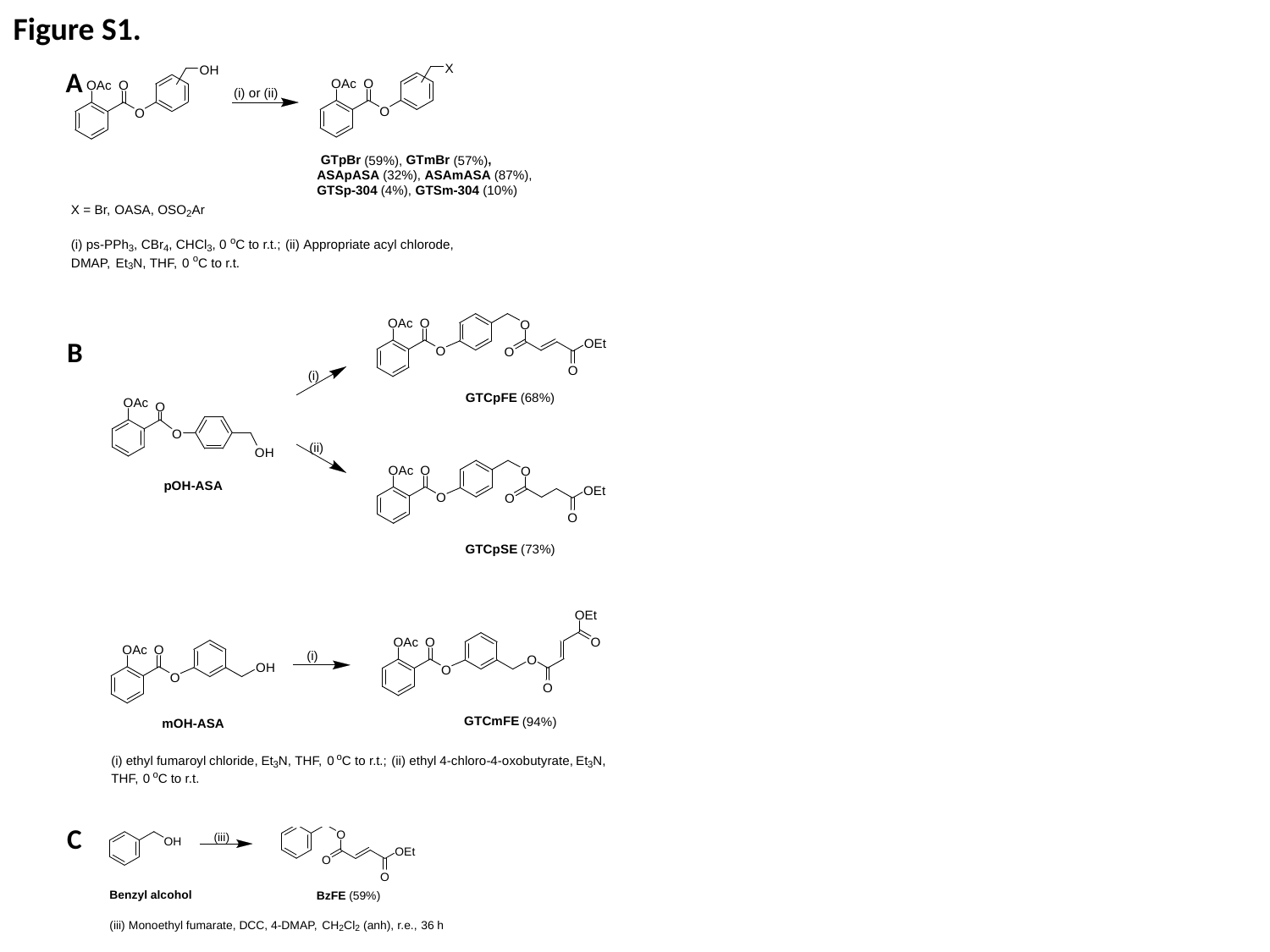

Figure S1.
A
B
C

Supplement: Additional file 2: Figure S1. — Synthesis of ASA prodrugs. Chemical structures and synthetic schemes are indicated and described in Additional file 1: Supplemental Methods. (PPTX 133 kb) [file 12885_2015_1868_MOESM2_ESM.pptx]

## Slide 1
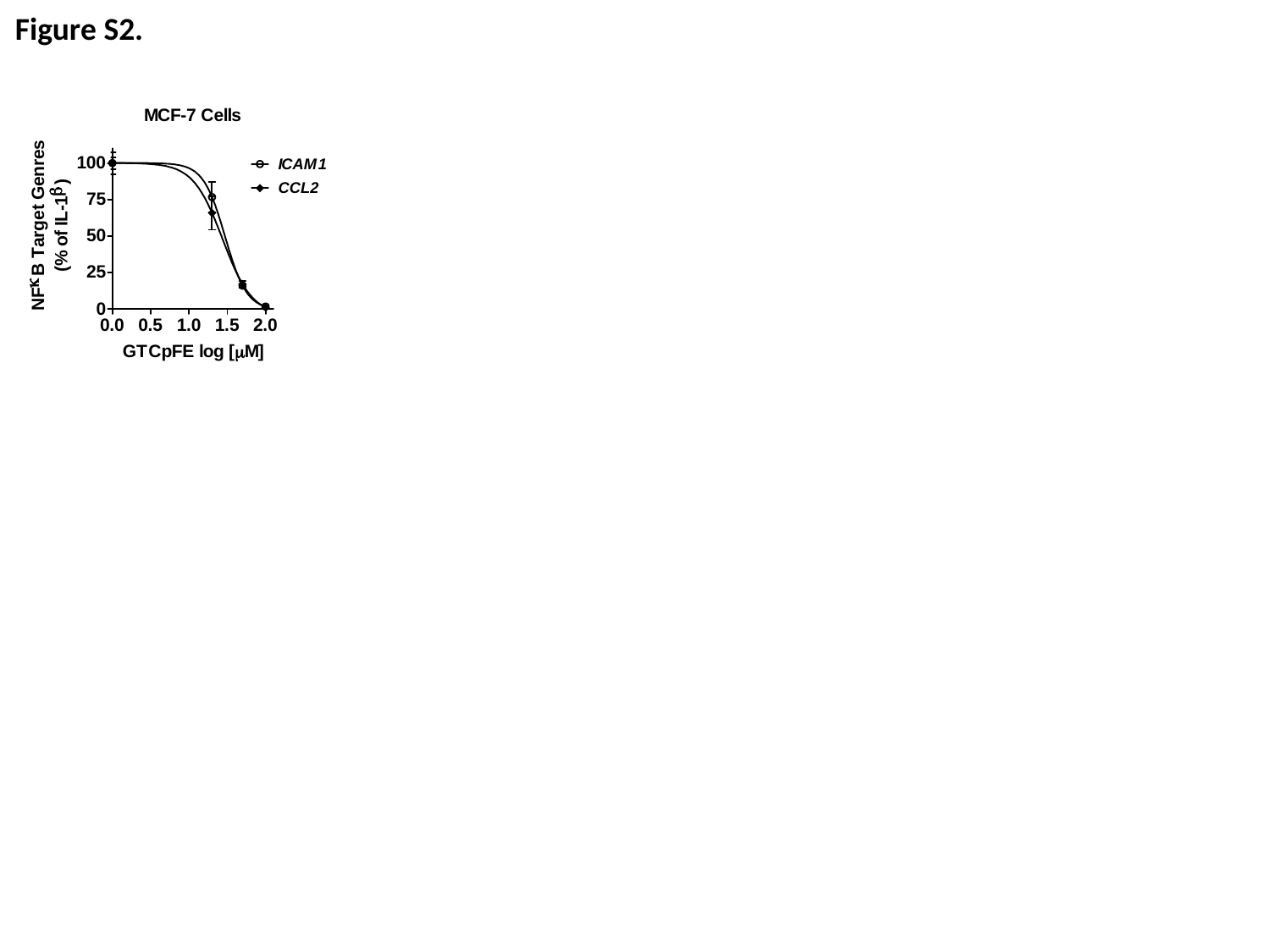

Figure S2.

Supplement: Additional file 3: Figure S2. — GTCpFE inhibits cytokine-induced NFκB target gene expression in breast cancer cells. MCF-7 cells were pretreated for 2 hours with increasing concentrations of GTCpFE followed by treatment with IL-1β (10 ng/ml) for another 2 hours. Expression of NFκB target genes, ICAM1 and CCL2 was measured by RT-QPCR. Drug inhibitory activity is plotted as % of IL-1β alone. (PPTX 72 kb) [file 12885_2015_1868_MOESM3_ESM.pptx]

## Slide 1
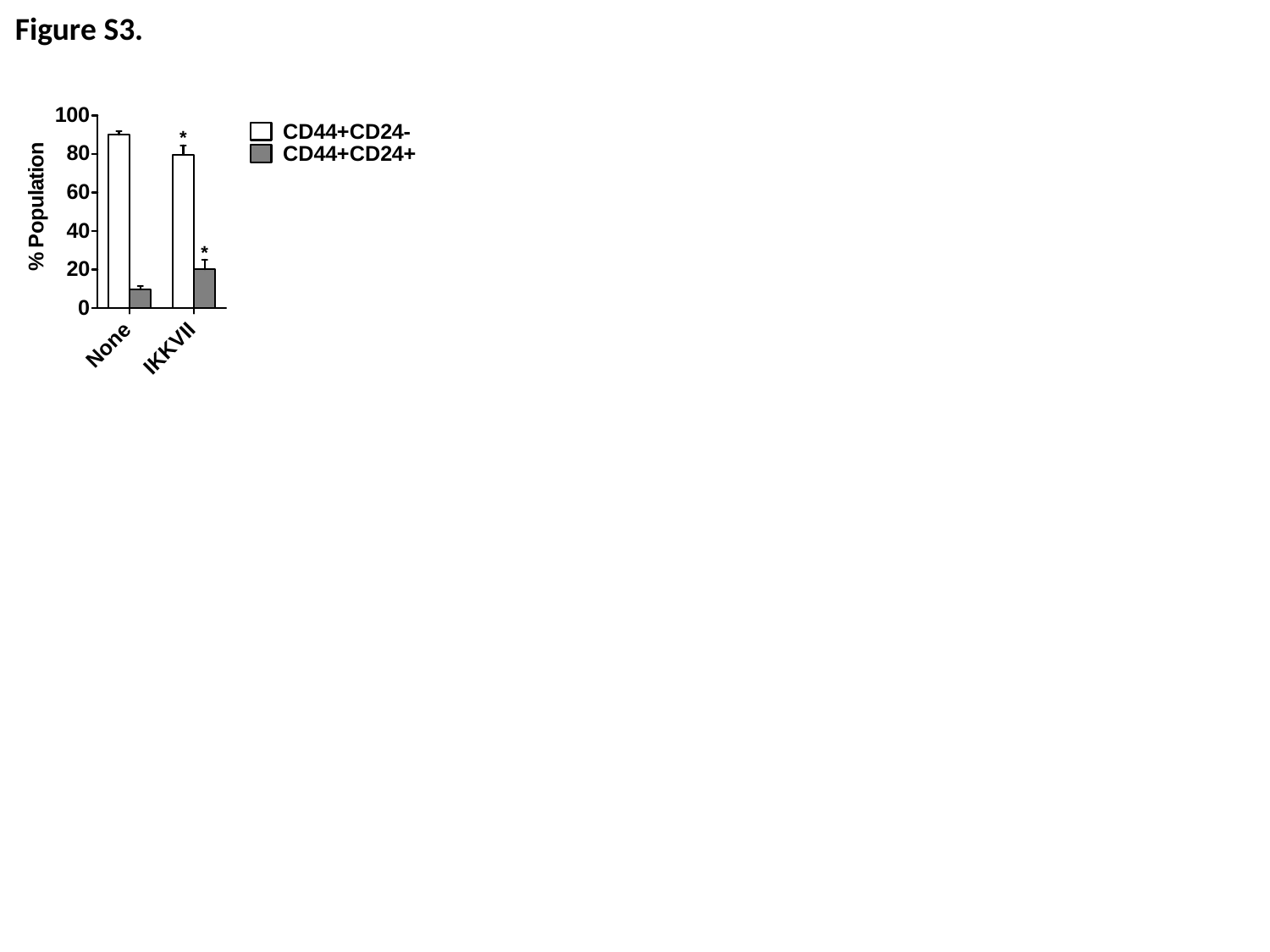

Figure S3.

Supplement: Additional file 4: Figure S3. — The effect of NFκB inhibitor, IKKVII, on the CD44+CD24− population. The CD44+CD24− population percentage was determined by FACS analysis of MDA-MB-231 cells treated with 2.5 μM IKKVII for 72 hours. * P < 0.05. (PPTX 48 kb) [file 12885_2015_1868_MOESM4_ESM.pptx]
